# Structural insight and stability of TNFR-Fc fusion protein (Etanercept) produced by using transgenic silkworms

**DOI:** 10.1093/jb/mvaa092

**Published:** 2020-08-07

**Authors:** Masato Kiyoshi, Ken-Ichiro Tatematsu, Minoru Tada, Hideki Sezutsu, Hiroko Shibata, Akiko Ishii-Watabe

**Affiliations:** 1 Division of Biological Chemistry and Biologicals, National Institute of Health Sciences, 3-25-26, Tonomachi, Kawasaki-ku, Kawasaki, Kanagawa 210-9501, Japan; 2 Transgenic Silkworm Research Unit, National Institute of Agrobiological Sciences National Agriculture and Food Research Organization, 1-2 Owashi, Tsukuba, Ibaraki 305-8634, Japan

**Keywords:** Fc fusion protein, glycosylation, physicochemical property, silkworm, therapeutic protein

## Abstract

Therapeutic proteins expressed using transgenic animals have been of great interest for several years. Especially, transgenic silkworm has been studied intensively because of its ease in handling, low-cost, high-yield and unique glycosylation patterns. However, the physicochemical property of the therapeutic protein expressed in transgenic silkworm remains elusive. Here, we constructed an expression system for the TNFR-Fc fusion protein (Etanercept) using transgenic silkworm. The TNFR-Fc fusion protein was employed to N-glycan analysis, which revealed an increased amount of afucosylated protein. Evidence from surface plasmon resonance analysis showed that the TNFR-Fc fusion protein exhibit increased binding affinity for Fcγ receptor IIIa and FcRn compared to the commercial Etanercept, emphasizing the profit of expression system using transgenic silkworm. We have further discussed the comparison of higher order structure, thermal stability and aggregation of the TNFR-Fc fusion protein.

## Graphical Abstract

**Figure:**
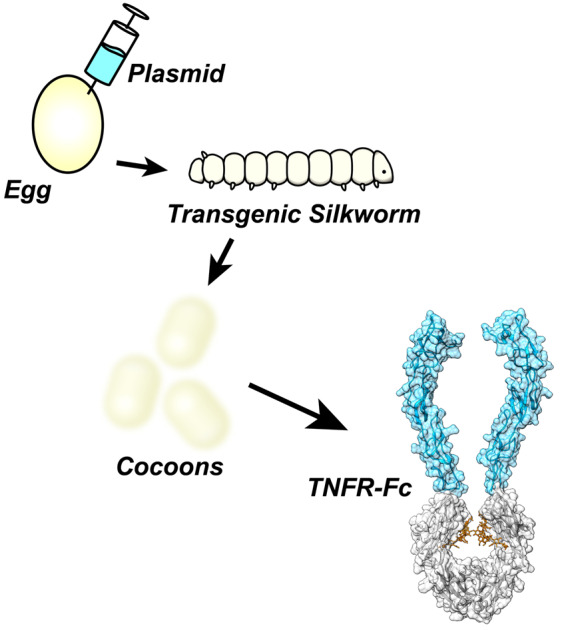

Therapeutic proteins belong to the most extensively growing class of drugs in recent years. Those therapeutic proteins have been approved for the treatments of a wide range of indications, from cancer, autoimmune diseases, to genetic disorders such as lysosomal storage diseases. Chinese hamster’s ovary (CHO) cells are frequently used as an expression systems, because (i) CHO cells produce high yield of therapeutic protein; (ii) characteristics of the protein including post-translational modifications are well studied and (iii) the methodology for protein expression is well-established (1, 2). However, the biotechnological development enables the use of transgenic animal, plant and insect cells as alternative expression systems for therapeutic proteins.

Transgenic silkworm (*Bombyx mori*) has been intensively focussed as an expressing system from the perspectives of ease in handling, low-cost, high-yield and unique glycosylation patterns (3–7). Recombinant canine interferon gamma and feline interferon gamma expressed using baculovirus-infected silkworms were approved as veterinary drugs (8, 9). Those cases emphasize the utility of silkworm-expression system.

The glycosylation pattern of antibody-Fc expressed in silkworms is known to be unique (6, 7). Most antibody-Fc derived from transgenic silkworm are carrying mannose-terminated glycans or less complete glycans compared to the native protein. As in the cases of therapeutic antibodies in which the glycosylation pattern of fragment Fc region of therapeutic antibody has a great impact on its stability and effector functions including antibody-dependent cell-mediated cytotoxicity (ADCC) and complement-dependent cytotoxicity (CDC), the glycosylation pattern of therapeutic proteins expressed in silkworm is quite important (10–13).

The physicochemical properties of therapeutic protein expressed in silkworm, including thermal stability, degradation and aggregation have not well-addressed so far. Here, we characterized the glycosylation pattern and physicochemical properties of therapeutic protein expressed in transgenic silkworm and compared their properties with those of native protein. In this study, we have used Etanercept as a model of therapeutic protein. Etanercept is the anti-tumor necrosis factor (TNF) therapeutic protein approved for the treatment of rheumatoid arthritis. Etanercept is a fusion protein, in which TNF receptor 2 (TNFR2) is fused to the human IgG1-Fc domain. TNFR-Fc fusion protein was successfully expressed in silkworm-expression system (termed as ‘sTNFR-Fc’). Glycan analysis revealed that the amount of afucosylated Fc was significantly higher in sTNFR-Fc compared to Etanercept. We employed structural and biological analysis. Furthermore, we focussed on their thermal stability and aggregation propensity in detail. This study certainly highlights the benefits of alternative expression system using transgenic silkworm for therapeutic protein.

## Materials and Methods

### Expression and preparation of TNFR2-Fc in transgenic silkworm (sTNFR-Fc)

**Fig. 1.**
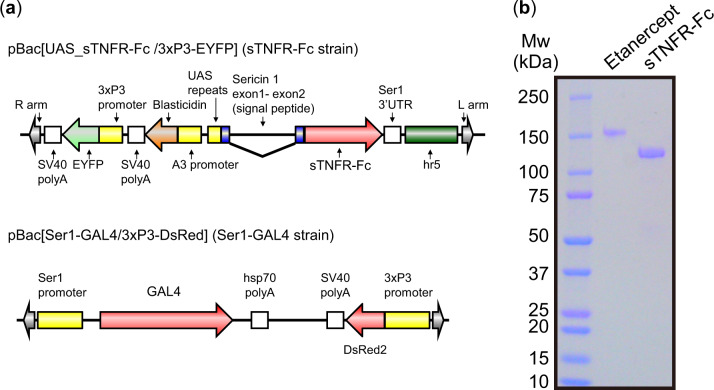
**Expression of sTNFR-Fc using transgenic silkworms.** (**a**) The structures of the plasmids used to generate transgenic silkworms. Each plasmid has right and left arms of *piggyBac* and the 3 × P3-fluorescent gene cassette for a screening marker (EYFP or DsRed2). pBac[UAS_sTNFR-Fc/3 × P3-EYFP] encodes sTNFR-Fc under the control of an UAS promoter and contains a BmNPV-derived hr5 enhancer and an A3-blasticidin cassette. The sTNFR-Fc gene was fused to the signal peptide sequence of the sericin1 gene. The plasmid pBac[Ser1-GAL4/3 × P3-DsRed] encodes the GAL4 gene under the control of the sericin1 promoter. (**b**) SDS–PAGE analysis of Etanercept and sTNFR-Fc. Etanercept (left) and sTNFR-Fc (right).

The amino acid sequence of sTNFR-Fc used in this study was identical to that of the commercial Etanercept (Enbrel^®^). To establish transgenic silkworm strain expressing sTNFR-Fc, we constructed pBac [UAS_sTNFR-Fc/3 × P3-EYFP] vector (Fig. 1) and injected the plasmid into silkworm eggs with helper plasmid DNA and mRNA that supply the *piggyBac* transposase. G0 adults were mated with other G0 adults potentially carrying the same plasmid to generate G1 eggs. G1 embryos were screened for expressions of EYFP in the eyes. To express sTNFR-Fc in the middle silk glands (MSGs) of transgenic silkworms, the sTNFR-Fc strain was mated with Ser1-GAL4 strain (Fig. 1) that expresses the GAL4 gene in MSGs (4). In the next generation, the transgenic silkworms that expressed both EYFP and DsRed2 in embryonic eyes were selected and used in the subsequent experiments.

The silkworm MSGs or cocoons were collected and suspended in phosphate-buffered saline (PBS), pH 7.2, containing 1% Triton X-100. The soluble fraction was subjected to a HiTrap Protein G HP column (GE Healthcare), which was pre-equilibrated with PBS. After washing with PBS, sTNFR-Fc was eluted with 0.1 M glycine–HCl (pH 3.0) and neutralized with 1.0 M Tris–HCl (pH 8.0). The concentration of protein was measured using a NanoDrop 2000c spectrophotometer (Thermo Fisher Scientific). The extinction coefficient at 280 nm of Etanercept and sTNFR-Fc is 0.8 ml/(mg cm), which were calculated based on the amino acid sequence.

### Surface plasmon resonance (SPR) analysis

SPR analysis was employed to Etanercept and sTNFR-Fc to compare their binding affinity for FcγRI, FcγRIIa, FcγRIIIa, FcRn and TNF. Biacore T200 (GE Healthcare) was used for the analysis. The C-terminus His-tagged ectodomain of FcγRs (FcγRI, FcγRIIa, FcγRIIIa) and TNF was obtained from Sino Biologicals and WAKO (Catalog # 207-15261), respectively. First, anti-human IgG antibody was immobilized onto the CM5 sensor chip. Etanercept or sTNFR-Fc was immobilized on the surface. Subsequently, binding sensorgrams were obtained by injecting each of the FcγRs and TNF at a flow rate of 30 μl/min. The interaction between FcRn and Etanercept/sTNFR-Fc was measured as described previously (14).

### Glycan analysis (LC/MS/MS)

N-glycosylation pattern of the Fc-part was analysed by peptide mapping using liquid chromatography/mass spectroscopy (LC/MS/MS), as described previously (6). Briefly, Etanercept and sTNFR-Fc dissolved in 0.5 M Tris–HCl, 8 M guanidine–HCl and 5 mM ethylenediaminetetraacetic acid (EDTA) (pH 8.6) was reduced with dithiothreitol and carboxy-methylated with sodium monoiodoacetamide. Desalted samples were employed for tryptic digestion (Promega) at 37°C for 4 h. The tryptic digests were dissolved in distilled water containing 2% acetonitrile and 0.1% trifluoroacetic acid. The samples were separated using an Eksigent Nano LC System (SCIEX) using a Nano LC column (3 μm, ChromXP C18CL; SCIEX). The mobile phase consisted of 0.1% formic acid in water (solvent A) and 0.1% formic acid in 90% acetonitrile (solvent B). The chromatography was performed with a gradient from 0% to 55% solvent B for 40 min at a flow rate of 0.3 ml/min. Mass spectrometric analyses were performed by using a TripleTOF 6,600 mass spectrometer (SCIEX). Mass spectra were acquired over *m*/*z* 400–2,000 for mass spectrometry (MS) and *m*/*z* 100–2,000 for MS/MS. The areas of the peaks were integrated to calculate the relative abundance of each N-glycan.

### Higher order structure analysis

The higher order structure of Etanercept and sTNFR-Fc was determined and compared using a sandwich array of anti-Etanercept peptide antibody (Enbrbridge; Array bridge Inc., St Louis, Missouri, USA) by following the instructions of the manufacturer. Etanercept and sTNFR-Fc at a concentration of 5 µg/ml were seeded into each well of a 96-well plates, which was coated with several antibodies which recognize the peptide of Etanercept. After incubation at room temperature for 1 h, the biotin-labelled reporting antibody was added. Subsequently, streptavidin-horseradish peroxidase (HRP) and tetramethylbenzidine substrate were used to detect the signal. Optical density at 450 nm was detected using an EnSpire Multimode Plate Reader (PerkinElmer). The measurements were performed in triplicate.

### Papain digestion and protein A purification

The Fc fragment of the Etanercept and sTNFR-Fc was obtained by papain digestion (Thermofisher cat# 44985). First, samples were desalted using Zeba Spin column. The pre-equilibrated and immobilized papain–resin and samples were incubated at 37°C for 12 h. Digested Fc and TNFR were purified with a protein A kit (Thermofisher cat# 44985) by following the instructions of the manufacturer.

### Differential scanning fluorimetry (DSF)

The thermal stability of Etanercept and sTNFR-Fc was determined by DSF analysis. The samples at a concentration of 0.5 μg/ml were mixed with the protein thermal shift dye (Applied Biosystems, Catalog # 4461146). The peaks of excitation and emission wavelength of the dye are 580 and 623 nm, respectively. A StepOne real-time polymerase chain reaction systems (Applied Biosystems) was programmed to increase the temperature from 25°C to 99°C at a scanning speed of 0.05°C/s.

### Accelerated protein degradation

Purified proteins were subjected to heat stress at 40°C and at 25°C (as a negative control) and acidic condition at pH 3.0 (50 mM citrate, 150 mM NaCl) and at pH 7.4 (PBS, as a negative control) for 1 month.

### Size exclusion chromatography (SEC)

Fifty micrograms of degraded samples was subjected to TSKgel G3000SWXL column (7.8 mm ID × 30 cm, 5.0 μm particle size, TOSOH) equilibrated with PBS, using ÄKTA Avant system (GE Healthcare). The flow rate was set at 0.5 ml/min, and the samples were analysed by ultraviolet absorbance at 280 nm.

## Results

### Generation of TNFR-Fc fusion protein using transgenic silkworm

**Fig. 2.**
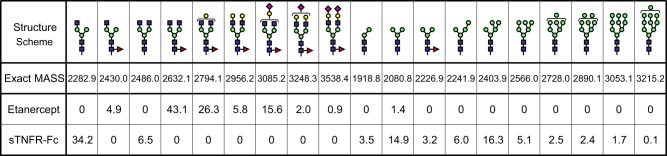
**N-glycosylation pattern of Fc-part.** Blue squares, green circles, yellow circles, magenta diamonds and red triangles correspond to GlcNAc, Man, Gal, sialic acid and Fuc, respectively. The relative abundance (%) of each N-glycan was shown.

The yield of sTNFR-Fc derived from MSGs and cocoons was 220 μg and 50 μg per worm, respectively. sTNFR-Fc derived from cocoons was used for subsequent analysis. Non-reducing sodium dodecyl sulfate polyacrylamide gel electrophoresis (SDS-PAGE) analysis of the Etanercept and sTNFR-Fc is given in Fig. 1b. Both proteins were of high purity. Approximately, protein bands of 150 and 125 kDa were detected. We have determined that the whole protein was expressed without lacking any domain using peptide mapping (data not shown). The difference in molecular weight between Etanercept and sTNFR-Fc indicated that the amount of glycosylation of TNFR-part of the sTNFR-Fc was decreased. Since a methodology for determining O-glycosylation patterns of TNFR-part of Etanercept is yet to be established so far, the O-glycosylation patterns of sTNFR-Fc remain elusive (15–17). Peptide mapping analysis determined the exact content of N-glycan attached to Asn297 of CH_2_ domain (Fig. 2). sTNFR-Fc exhibited more reduced level of fucosylation (7). The relative abundance of fucosylated protein in Etanercept and sTNFR-Fc was 98.6% and 3.2%, respectively. The relative abundance of high-mannose glycans in Etanercept and sTNFR-Fc was 1.4% and 55.7%, respectively.

### Binding affinity for FcγRs, FcRn and TNF

**Fig. 3.**
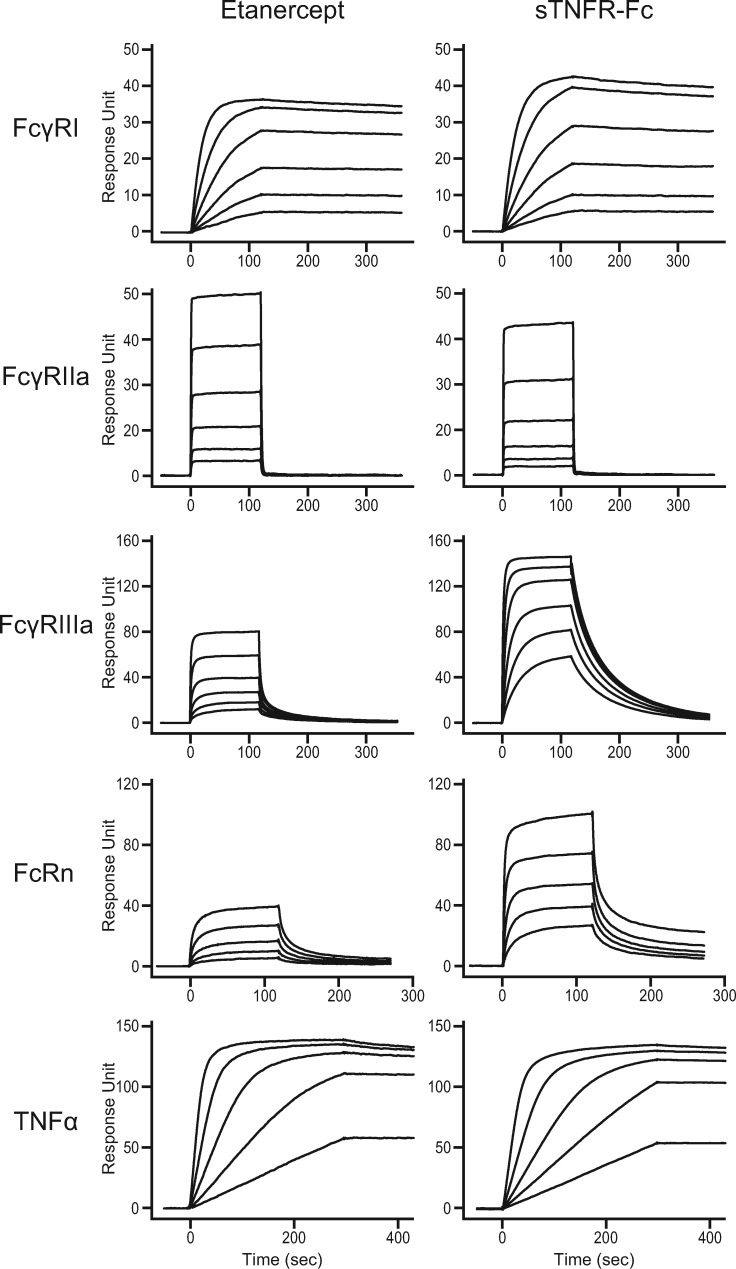
**Binding sensorgrams of Etanercept and sTNFR-Fc examined using SPR.** Every experiment was run in at least five different concentrations.

Since the interaction between Fc and FcγRs has a profound impact on multifaceted properties, such as effector functions which are elicited by several immune cells, SPR analysis was employed to quantify the affinity of Etanercept/sTNFR-Fc for FcγRs (FcγRI, FcγRIIa, FcγRIIIa), FcRn and TNF. Note that the main mechanism of Etanercept is the inhibition of TNF by neutralizing and not ADCC nor CDC. The sensorgrams of Etanercept and sTNFR-Fc for the binding with those proteins, which are immobilized on a sensor chip, are given in Fig. 3. The values of dissociation constant (*K_D_*) calculated from a fitting curve are given in Table I. The affinity of sTNFR-Fc towards FcγRI and FcγRIIa is slightly decreased compared to that of Etanercept (59% and 44%, respectively). On the other hand, the affinity of sTNFR-Fc towards FcγRIIIa is significantly increased. The affinity of sTNFR-Fc for FcγRIIIa was 4.4-fold higher than that of Etanercept. This affinity improvement was assumed to be associated with the lower relative abundance of fucosylated protein in sTNFR-Fc.

**Table I.** Binding affinity of Etanercept and sTNFR-Fc for FcγRs, FcRn and TNF

Protein	*K_D_* (M)		*K_D_* (Etanercept)/*K_D_* (sTNFR-Fc) (affinity increase relative to Etanercept)
	Etanercept	sTNFR-Fc	
FcγRI	1.6 ± 0.2 × 10^−10^	2.7 ± 0.7 × 10^−10^	0.6
FcγRIIa	2.6 ± 0.04 × 10^−7^	5.9 ± 0.02 × 10^−7^	0.4
FcγRIIIa	7.4 ± 0.7 × 10^−8^	1.7 ± 0.03 × 10^−8^	4.4
FcRn	2.5 ± 0.4 × 10^−6^	1.1 ± 0.02 × 10^−6^	2.7
TNF	3.7 ± 0.4 × 10^−10^	3.6 ± 0.4 × 10^−10^	1.0

The Fc region of therapeutic protein binds to the salvage receptor (FcRn), leading to long pharmacokinetic half-life *in vivo*; hence, the molecular interaction between Fc-FcRn has been studied intensively (18). As shown in Table I, sTNFR-Fc exhibited 2.3-fold higher affinity for FcRn compared to Etanercept (*K_D_*_Etanercept_; 2.5 μM, *K_D_*_sTNFR-Fc_; 1.1 μM). sTNFR-Fc and Etanercept have nearly the same affinity for TNF.

### Structural perspective

**Fig. 4.**
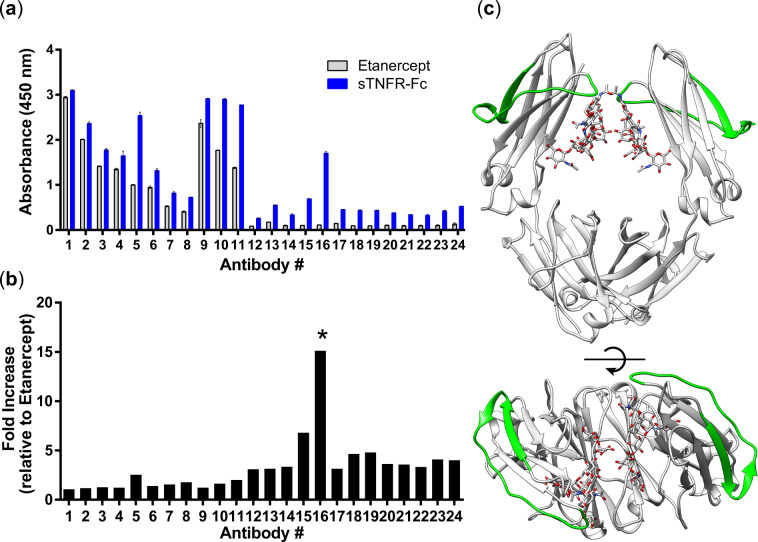
**Structural comparison examined using antibody array.** (**a**) Detected absorbance of each antibodies. Grey bar: Etanercept and blue bar: sTNFR-Fc. Error bar indicates standard deviation of three replicates. (**b**) Value of fold increase of sTNFR-Fc. The value of sTNFR-Fc was divided by the value of Etanercept. The asterisk highlights the antibody # 16. (**c**) The peptide that antibody # 16 recognizes is depicted green in crystal structure of Fc. Protein Data Bank ID: 4W4N.

Antibody array (ELISA) is a prominent methodology that gives information on specific conformational differences between two therapeutic proteins (19). The array provides detailed insight into conformational comparability using antibodies that recognize disordered peptides of Etanercept. Each antibodies # from 1 to 11 recognize a peptide of TNFR2 part (residues ranging from 1 to 238 of the amino acid sequence) and antibodies # from 12 to 24 recognize a peptide of Fc-part (residues ranging from 215 to 451 of the amino acid sequence). As shown in Fig. 4a, sTNFR-Fc represented higher values in all regions (antibody # from 1 to 24) compared to Etanercept. Notably, the peptide of Fc-part of sTNFR-Fc exhibited remarkably high value indicating that the Fc-part of sTNFR-Fc is highly perturbed in solution. Particularly, antibody # 16 (corresponds to the peptide ranging from V288 to S313) showed extremely high value compared to Etanercept (15-fold increase) (Fig. 4b). The peptide is located at exactly the N-glycosylation site of the CH_2_ domain (Fig. 4c).

### Thermal stability

**Fig. 5.**
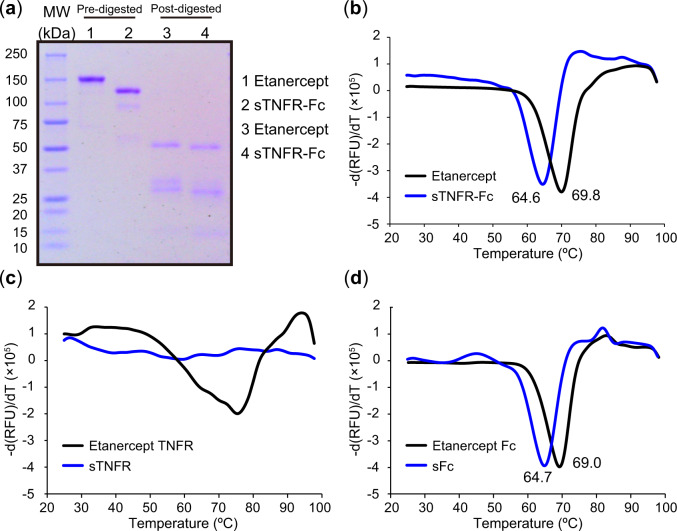
**SDS–PAGE analysis and denaturation curve examined by DSF method.** (**a**) SDS–PAGE analysis; (**b**) black: Etanercept, blue: sTNFR-Fc; (**c**) black: Etanercept TNFR, blue: sTNFR; (**d**) black: Etanercept Fc, blue: sFc.

Protein stability was examined using DSF. Both proteins exhibited single denaturation peak, corresponds to melting temperature (Fig. 5b). The value of melting temperature for Etanercept and sTNFR-Fc was found to be 69.8°C and 64.6°C, respectively, indicating that the sTNFR-Fc is thermally less stable. To identify the difference of thermal stability is derived from TNFR2-part, or Fc-part, both proteins were subjected to papain digestion and protein A purification (Fig. 5a). The proteins separated into TNFR2-part and Fc-part were further examined their thermal stability using DSF (Fig. 5c and d). As shown in Fig. 5c, the peaks were not defined clearly indicating the hydrophobicity of TNFR2-part. Especially, the denaturation curve of sTNFR-part was hardly measurable. In contrast, Fc-part exhibited a single denaturation peak (Fig. 5d). The value of melting temperature of the Fc derived from Etanercept and sTNFR-Fc was found to be 69.0°C and 64.7°C, respectively. These results demonstrated that the difference of thermal stability of Etanercept and sTNFR-Fc was primarily derived from the Fc-part but the difference of thermal stability at TNFR-part was also revealed.

### Accelerated (forced) degradation

It is widely accepted that there is a strong correlation between Fc-glycosylation and long-term stability of therapeutic proteins (20–24). The accelerated degradation study provides information on the potential long-term storage stability (25). Exposure of therapeutic proteins to harsh environments results in various degradation pathways, such as denaturation, aggregation and fragmentation.

**Fig. 6.**
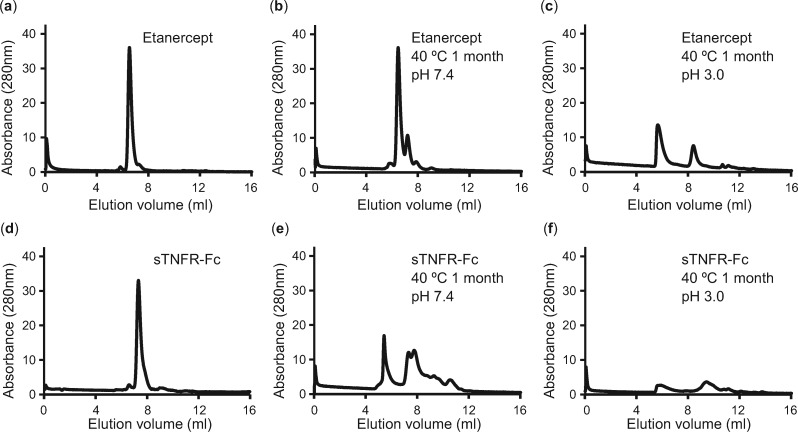
**SEC chromatogram of degraded proteins.** (**a**) Native Etanercept. (**b**) Etanercept degraded in 40°C for 1** **month at pH 7.4. (**c**) Etanercept degraded in 40°C for 1 month at pH 3.0. (**d**) Native sTNFR-Fc. (**e**) sTNFR-Fc degraded in 40°C for 1 month at pH 7.4. (**f**) sTNFR-Fc degraded in 40°C for 1 month at pH 3.0.

The propensity of denaturation, aggregation and fragmentation of Etanercept and sTNFR-Fc was compared by exposing harsh stresses as follows: (1) thermal; at 40°C and (2) acidic condition; at pH 3.0 and pH 7.4 (as a negative control) for 1 month. The samples were subjected to SEC analysis to examine aggregation and fragmentation. Etanercept showed a single peak corresponding to monomeric protein (Fig. 6a). After degradation at pH 7.4, whereas peaks of oligomer and fragments appeared, the peak of monomer remained as the major component (Fig. 6b). After degradation at pH 3.0, the peak of monomer disappeared because proteins were fragmented and aggregated in the acidic condition (Fig. 6c). Compared to Etanercept, sTNFR-Fc showed higher aggregation rate after degradation at pH 7.4 (Fig. 6e). Similar to Etanercept, no monomeric peak was observed after degradation at pH 3.0 (Fig. 6f).

**Fig. 7.**
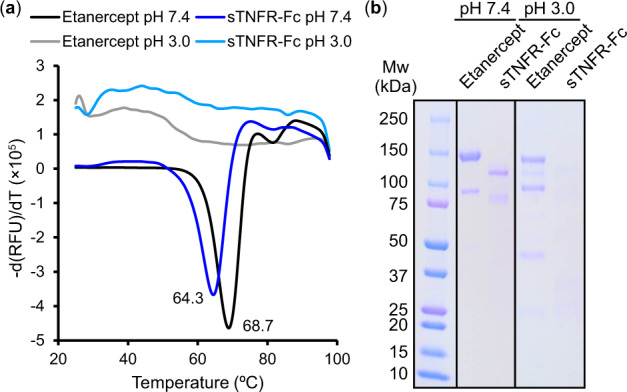
**DSF and SDS–PAGE analysis of degraded Etanercept and sTNFR-Fc.** (**a**) Black: Etanercept degraded in 40°C for 1 month at pH 7.4. Blue: sTNFR-Fc degraded in 40°C for 1 month at pH 7.4. Grey: Etanercept degraded in 40°C for 1 month at pH 3.0. Light blue: sTNFR-Fc degraded in 40°C for 1 month at pH 3.0. (**b**) Left: degraded in 40°C or 1 month at pH 7.4, right: degraded in 40°C for 1 month at pH 3.0.

The degraded samples were further analysed using DSF and SDS–PAGE. Consistent with the results of SEC analysis, Etanercept and sTNFR-Fc degraded at pH 3.0 generated no clear denaturation curve (Fig. 7a). The SDS–PAGE analysis confirmed that the degraded proteins at pH 3.0 were largely fragmented. Particularly, sTNFR-Fc degraded at pH 3.0 represented only a blurred protein band (Fig. 7b).

## Discussion

There has been a strong interest of expressing therapeutic proteins in various host cells, such as animal, plant or insect cells from perspectives of lowering cost, enhanced effector function. Given that there is a strong need to accumulate studies of characterization of the effector function and physicochemical property. Here, we have focussed on transgenic silkworm as an alternative expression system, owing to the ease in handling, low-cost and unique glycosylation patterns. In this study, Etanercept was adopted as a model therapeutic protein.

Thus far, to the best of our knowledge, this is a first example of the study for characterization of Fc fusion protein expressed in transgenic silkworm. Successfully expressed sTNFR-Fc fusion protein exhibited unique N-glycosylation patterns in the Fc-part. The decreased content of fucosylated protein of sTNFR-Fc was consistent with the previous study of monoclonal antibody expressed in MSGs or cocoons of transgenic silkworm (7, 26). Iizuka *et al.* showed that the content percentage of fucosylated protein expressed in MSGs or cocoons is significantly low compared to other organs (fat body, haemolymph, etc.). Decreased content of fucosylated protein resulted in increased affinity for FcγRIIIa. On the other hand, the affinity of sTNFR-Fc for TNF was unchanged compared to Etanercept confirming the advantage of using transgenic silkworm as a host for expressing therapeutic protein.

SDS–PAGE analysis showed that the molecular weight of sTNFR-Fc was smaller than Etanercept. This is probably because the TNFR-part was less O-glycosylated. Determining O-glycosylation of TNFR-part is quite important for understanding the physicochemical property of sTNFR-Fc.

The antibody array analysis shed light on the specific peptide ranging from V288 to S313, which is extremely disordered region. The peptide is exactly located at the position where the N-glycan is covalently connected. Also, the peptide is exactly located at the C’E loop, where Subedi and Barb have previously discussed that the motion is increased upon the absence of N-glycan (27). The shorter N-glycan makes the Fc protein more perturbed because of less interactions between the N-glycan and the Fc protein. This phenomenon has been demonstrated by multiple studies (27–31). Especially, Yamaguchi *et al.* showed that the number of perturbed residues in the Fc is increased upon step-wise cleavage of the N-glycan using NMR (32). In our study, the N-glycan of sTNFR-Fc is relatively shorter than that of Etanercept; probably, this is because why the peptide of sTNFR-Fc is more disordered compared to Etanercept. These observations are in good agreement with the results Yamaguchi *et al.* showed.

Given the structural characteristics, we hypothesized that sTNFR-Fc could easily to be denatured and thermally less stable compared to Etanercept. Our previous study showed that the anti-HER2 monoclonal antibodies (Trastuzumab) produced by the silkworm baculovirus expression system was thermally less stable compared to commercial Trastuzumab (6). Indeed, DSF, SEC and SDS–PAGE analysis illustrated that the sTNFR-Fc was fragmented in physiological condition and in acidic condition. The DSF analysis revealed that the Tm of Fc-part of sTNFR-Fc was 4.3°C lower than Fc-part of Etanercept. When looking at TNFR-part, we found the TNFR-part exhibited no clear denaturation signal due to the decreased glycosylation. Those results certainly help estimating the pharmacological effect of the therapeutic protein expressed using silkworm.

In summary, we revealed that the TNFR2-Fc fusion protein expressed by silkworm is profitable, but thermally unstable, owing to the decreased glycosylation of TNFR-part and the highly disordered specific peptide. When transgenic silkworm is used to express a therapeutic protein in which there are multiple glycosylation sites, the profound focus is necessary to consider the decreased stability due to different glycosylation patterns. Accumulating studies on the correlation between N-glycosylation and the characteristics of therapeutic protein is quite significant (12, 33). The present study aids in advance the use of those transgenic animal-derived therapeutic proteins.

## Author contributions

M.K., T.K. and M.T. designed the experiments. M.K., T.K., T.M., S.H. and A.I. conducted the research and analysed the data. All authors contributed to the preparation and discussion of the manuscript.

## Abbreviations

ADCC: antibody-dependent cell-mediated cytotoxicity
CDC: complement-dependent cytotoxicity
CHO: Chinese hamster’s ovary
DSF: differential scanning fluorimetry
LC/MS/MS: liquid chromatography/mass spectroscopy
MS: mass spectrometry
MSGs: middle silk glands
PBS: phosphate-buffered saline
SEC: size exclusion chromatography
SPR: surface plasmon resonance
TNF: anti-tumor necrosis factor
TNFR2: TNF receptor 2.
